# A Second Generation Mn-Porphyrin Dimer with a Twisted Linker as a Potential Blood Pool Agent for MRI: Tuning the Geometry and Binding with HSA

**DOI:** 10.3390/ph13100282

**Published:** 2020-09-29

**Authors:** Hanlin Liu, Weiran Cheng, Shili Dong, David Feng Xu, Keith Tang, Xiao-an Zhang

**Affiliations:** 1 Department of Chemistry, University of Toronto, 80 St. George Street, Toronto, ON M5S 3H6, Canada; hanlin.liu@mail.utoronto.ca (H.L.); weiran.cheng@mail.utoronto.ca (W.C.); keithh.tang@mail.utoronto.ca (K.T.); 2 Department of Physical and Environmental Sciences, University of Toronto Scarborough, 1265 Military Trail, Toronto, ON M1C 1A4, Canada; shili.dong@mail.utoronto.ca (S.D.); davidfeng.xu@mail.utoronto.ca (D.F.X.); 3 Department of Biological Sciences, University of Toronto Scarborough, 1265 Military Trail, Toronto, ON M1C 1A4, Canada

**Keywords:** MRI contrast agents, blood pool agent, manganese porphyrin, human serum albumin

## Abstract

Blood-pool agents (BPAs) are MRI contrast agents (CAs) characterized by their long circulation in the vascular system to provide an extended time window for high-resolution MR angiography (MRA). Prolonged vascular retention, however, impedes the excretion of BPAs. Therefore, chemical strategy to regulate the balance between retention and clearance is important to reach optimal pharmacokinetics. We recently developed MnP2, the first Mn(III)-porphyrin (MnP) based BPA. MnP2 shows high *T*_1_ relaxivity (*r*_1_) and high affinity to human serum albumin (HSA) that leads to up to 48-h vascular retention in rats. However, upon albumin binding, the *r*_1_ is decreased. To modulate vascular retention time and plasma *r*_1_, a regioisomer of MnP2, *m*-MnP2, was synthesized. The free *m*-MnP2 exhibits lower *r*_1_ than that of MnP2 at magnetic fields above 2 MHz, which agrees with their relative hydrodynamic sizes. The HSA binding of *m*-MnP2 was evaluated using UV-Vis spectroscopy and found to have tuned-down affinity in comparison with MnP2. Upon HSA binding, the protein complex of *m*-MnP2 exhibits an *r*_1_ of 11.8 mM^−1^ s^−1^ at 3 T, which is higher than that of MnP2 bound to HSA. Taken together, this demonstrated the role of molecular geometry in optimizing the pharmacokinetics of albumin-targeting BPAs.

## 1. Introduction

Developed for magnetic resonance angiography (MRA) [1], blood-pool agents (BPAs) are used to provide anatomical and functional information of the vascular system and to diagnose vascular related diseases such as atherosclerosis and hemorrhage [2]. The only FDA approved BPA is Gadofosveset (formerly MS-325), a gadolinium (Gd)-based contrast agent (GBCA). Gadofosveset is designed to bind non-covalently to HSA [3,4], the most abundant carrier protein in the blood, for prolonged blood circulation and increased relaxivity. GBCAs are, however, associated with a severe adverse effect known as nephrogenic systemic fibrosis (NSF) in patients with kidney dysfunction. The in vivo deposition of Gd ions found in NSF subjects by post-mortem analysis implies that the high thermodynamic stability of Gd complexes in vitro is compromised in vivo when renal clearance is impeded, especially for GBCAs with linear chelates [5]. More recently, cases of Gd-deposit in the brain were increasingly reported, even in patients with healthy kidneys [6,7]. Thus, the main concern of using Gd-based BPAs for MRA is the increased risk of toxic Gd(III) ion release and accumulation due to the long vascular retention. Moreover, due to poor sales, Gadofosveset was withdrawn from the market. Therefore, a next generation BPA with improved efficacy and safety profile is demanded to fill the gap. To avoid risk of Gd-toxicity completely, Gd-free MRI CAs with high relaxivity are desired.

We chose manganese(III) porphyrins (MnPs) to construct non-Gd BPAs because of their biocompatibility, high stability, high relaxivity especially at high magnetic fields, and versatility for structural modulation [8,9,10,11,12]. Unlike Gd, which is a rare earth element exogenous to human body, Mn is an essential micronutrient and as such is more biocompatible than Gd. Furthermore, upon metal insertion into the rigid porphyrin macrocycle, the Mn(III) complex formed is kinetically inert and thermodynamically stable against metal dissociation, thanks to the strong metal-ligand interaction. In comparison, Gd(III) complexes are kinetically labile, due to poor orbital overlap between the *f*7 metal ion and the organic ligand. In terms of relaxivity, certain water-soluble MnPs were seen to exhibit significantly higher *T*_1_ relaxivity (*r*_1_) compared to GBCAs of comparable molecular weight [8]. Finally, *meso*-substituted porphyrin chemistry is well known for the versatile synthetic methods established during the past few decades, allowing for feasibility and flexibility for structural modifications. An array of new MnPs have been designed and constructed for various specific purposes, including extracellular [10], intracellular [9], tumour-targeting [12], and molecular sensing [11] applications.

A dimeric MnP, MnP2, was recently developed by our group and was found to exhibit anomalously high *r*_1_ at clinical magnetic field strengths ranging from 1~3 T [8]. In principle, a high field is the preferred condition for MRA to improve the S/N ratio, resolution and scan time [13]. At 3 T, the molar *r*_1_ of MnP2 in the form of the HSA complex was found to be twice that of Gadofosveset-HSA. Interestingly, however, the *r*_1_ of MnP2 is slightly decreased upon HSA binding, suggesting that MnP2 may be buried in the hydrophobic protein pocket, hindering water access to the Mn(III) ion [14]. The hydrophobic biphenyl linker of MnP2 contributes to strong HSA binding, resulting in a significantly prolonged vascular circulation, relative to other MnPs, such as Mn(III) *meso*-tetra(4-sulfonato-phenyl)porphine (MnTPPS) and Gd-based CAs [14,15,16].

**Figure 1:**
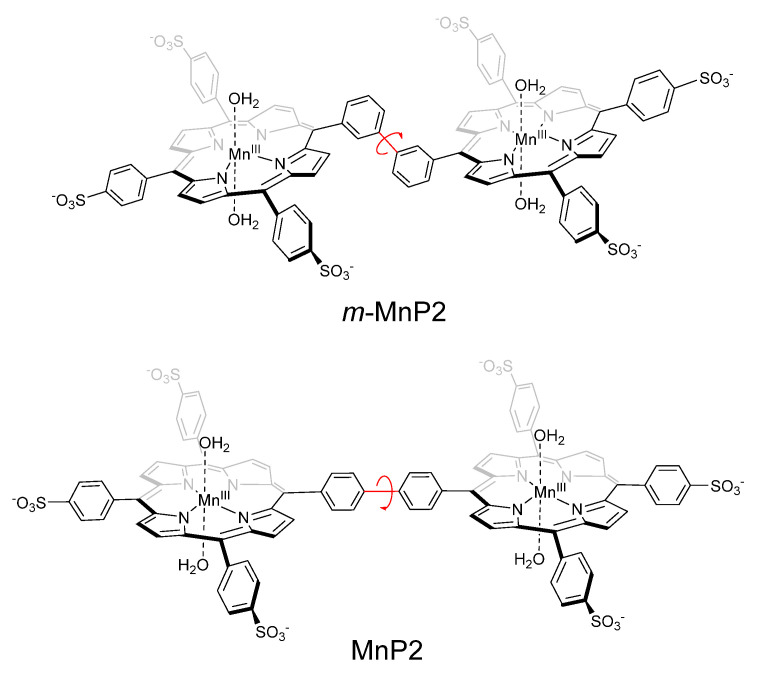
Chemical structure of *m*-MnP2 (**top**) designed with a biphenyl bridge connected at the *meta*-positions. MnP2 (**bottom**) is connected at the *para*- position of the biphenyl.

The long-circulating MnP2 opens the possibility of continuous longitudinal MRA with a single BPA injection. Some MRA applications, however, can be completed relatively quickly if further monitoring is not required. Rapid clearance can reduce in vivo exposure to exogenous CAs and therefore lower the risk of side-effects. This is crucial for GBCAs since insufficient renal clearance is known to cause NSF. Even though the MnP-based CAs are free of Gd toxicity, a chemical approach to adjust the balance between retention and clearance is valuable, as modern applications of MRA are rapidly diversifying. To accommodate the various criteria of different MRA applications, we anticipated that by tuning the binding interaction between MnP and HSA, the vascular retention of the MnP can be controlled. Since the biphenyl linker (Figure 1) of MnP2 is believed to be the major binding domain for HSA, perturbation of the biphenyl unit will change how the MnP interacts with the protein. Furthermore, modulation of the binding with HSA may avoid the water hindrance that is assumed in the MnP2–HSA complex [14] and thereby improve plasma *r*_1_ of the MnP. In this article, a new dimeric MnP, *m*-MnP2 with a twisted biphenyl linker (Figure 1), was designed, synthesized, and characterized.

## 2. Results and Discussion

### 2.1. Synthesis

**Scheme 1:**
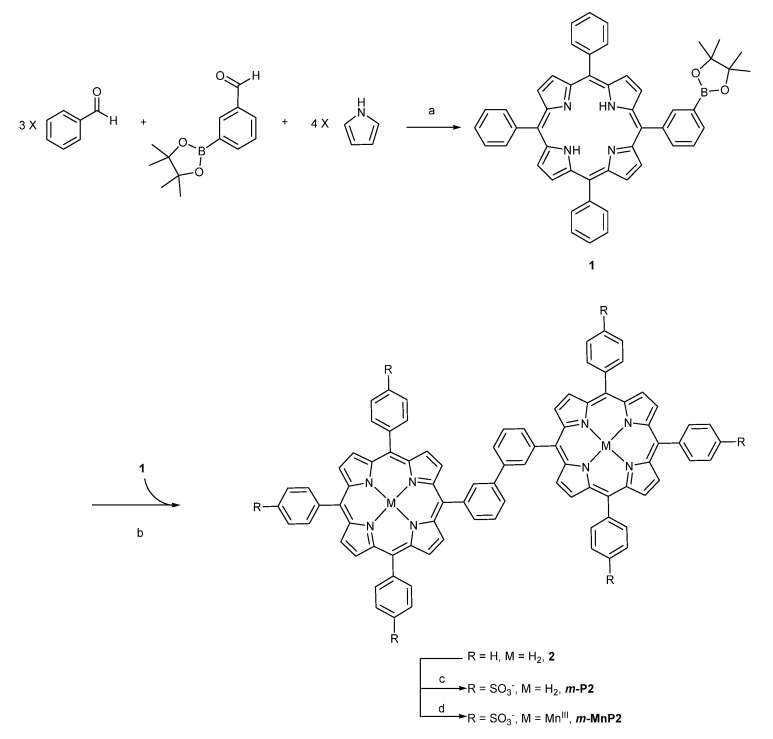
(**a**) BF_3_·Et_2_O, CH_2_Cl_2_, DDQ, rt, 18% (**b**) 1, Pd(PPh_3_)_2_Cl_2_, TBAF, H_2_O/THF, rt, 56%; (**c**) conc. H_2_SO_4_, 55˚C, 36%; (**d**) Mn(OAc)_2_, DMF, reflux, 91%.

The synthesis of *m*-MnP2 (Scheme 1) followed a similar pathway to that of MnP2 [8]. The monomeric porphyrin building block **1** was synthesized using a mild acid catalyzed condensation reaction between aldehydes and pyrrole [17]. Dimerization of 1 via a palladium catalyzed homolytic coupling reaction produced 2 at 56% yield [18]. Taking advantage of the low solubility of 2, the intermediate was purified by multiple washes of 40/60 CH_2_Cl_2_/hexanes and diethyl ether with repeats of centrifugation followed by sonication until the organic solvent was colourless upon centrifugation. Purity of 2 was solely confirmed by TLC due to low solubility. Sulfonation of 2 was carried out using concentrated sulfuric acid. Because the starting material 2 is insoluble in water, the progress of the reaction could be monitored with UV-visible absorption at 422 nm of the aqueous aliquot, indicating the graduate formation of the polar and water-soluble product *m*-P2 in solution. Unlike the previously synthesized P2 [8], 2′s reactive *para*-position of the biphenyl ring is open to possible sulfonation side reaction. Therefore, careful control of the reaction conditions were required. To minimize under- or over-sulfonation (Figure S8), the optimal conditions was found to be 6 h under 55 °C, yielding the most desired product (36%), eluting at 5.8–7 min on reverse phase HPLC using an Agilent Eclipse XDB-C18 4.6 × 150 mm (5 μm particle size) column using a gradient sequence 0–8 min: linear gradient from 26% to 30% acetonitrile; 8–9 min: linear gradient from 30% to 50% acetonitrile; 9–12 min: constant 50% acetonitrile at a flow rate of 0.8 mL/min. A reverse phase automated flash chromatography system was used to purify *m*-P2, characterized by ^1^H-NMR and ESI-MS (see Supporting Information). Manganese insertion in the presence of air and the organic base *N*,*N*-Diisopropylethylamine (DIPEA) yielded the final product *m*-MnP2, which was filtrated using ion exchange chromatography to remove excess Mn(II) salts. The UV-visible absorption spectrum of *m*-MnP2 (Figure S6) is similar to that of MnP2, with a Soret band at 468 nm and five characteristic peaks at 380, 402, 422, 568, and 602 nm, which are typical features of Mn(III) porphyrins [8,19].

### 2.2. Relaxometry Characterization

**Figure 2:**
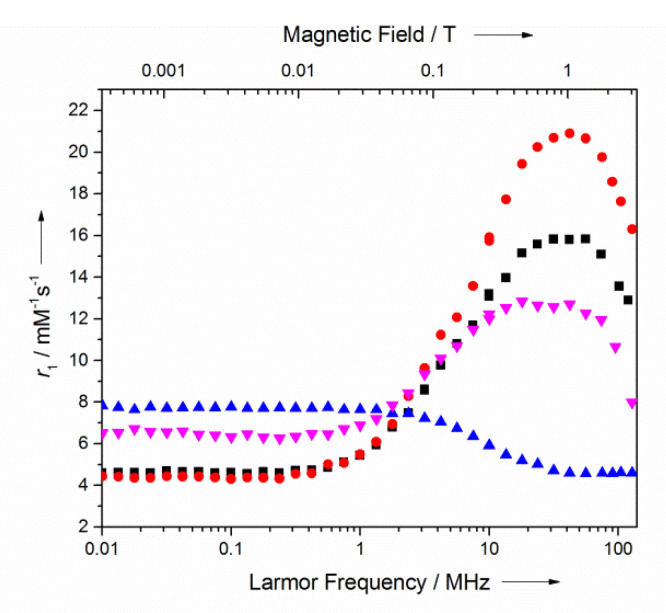
The nuclear magnetic relaxation dispersion (NMRD) profiles of Gd-DTPA (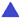), MnTPPS (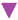), *m*-MnP2 (⬛), and MnP2(🔴) in 25 mM pH 7.2 HEPES buffer from 0.23 mT to 3 T at 25 °C. MnP *r*_1_ was normalized to per Mn(III) ion.

To investigate the field-dependent MRI relaxation enhancement efficiency of *m*-MnP2, its nuclear magnetic relaxation dispersion (NMRD) profile was acquired on a SMARTracer, coupled with an HTS-110 NMR system covering magnetic fields up to 3 T (Figure 2). The monomeric MnP building block, MnTPPS, as well as Gd-DTPA, a classic clinical GBCA, were chosen as control references for comparison.

All the MnP CAs exhibit higher *r*_1_ than Gd-DTPA at 1 T, demonstrating their unique strength of high relaxivities at high clinical fields. The fully extended MnP2 attains its *r*_1_ peak close to 1 T (41.8 mM^−1^ s^−1^ or 20.9 mM^−1^ s^−1^ per Mn). The intermediate relaxivity peak of *m*-MnP2 (*r*_1_ = 31.6 mM^−1^ s^−1^ or 15.8 mM^−1^ s^−1^ per Mn) at 1 T lying in between that of MnTPPS (*r*_1_ = 12.8 mM^−1^ s^−1^) and MnP2 is in agreement with its relative molecular size (Figure S7). This *r*_1_ trend is consistent with the predicted hydrodynamic sizes, indicating that decreases in molecular tumbling rate of MnPs enhance the *r*_1_.

As summarized in Table 1, for both MnP dimers, the high *r*_1_ peaks are sustained at higher fields up to 3 T with a moderate decrease, favouring applications at high clinical magnetic fields. As a pair of constitutional isomers, the molecular weight of *m*-MnP2 and MnP2 are exactly the same. As shown in Figure 1, the rotation of single bond in the middle of the biphenyl unit does not cause a dramatic change on the hydrodynamic size of MnP2, but can lead to a significant decrease to the hydrodynamic size of *m*-MnP2, as illustrated by computational molecular modeling (Figure S7). Our results demonstrated that the slight change in MnP connection at the biphenyl bridge resulted in a significant impact on molecular geometry and thus on the *r*_1_.

### 2.3. Binding Affinity with HSA

**Figure 3:**
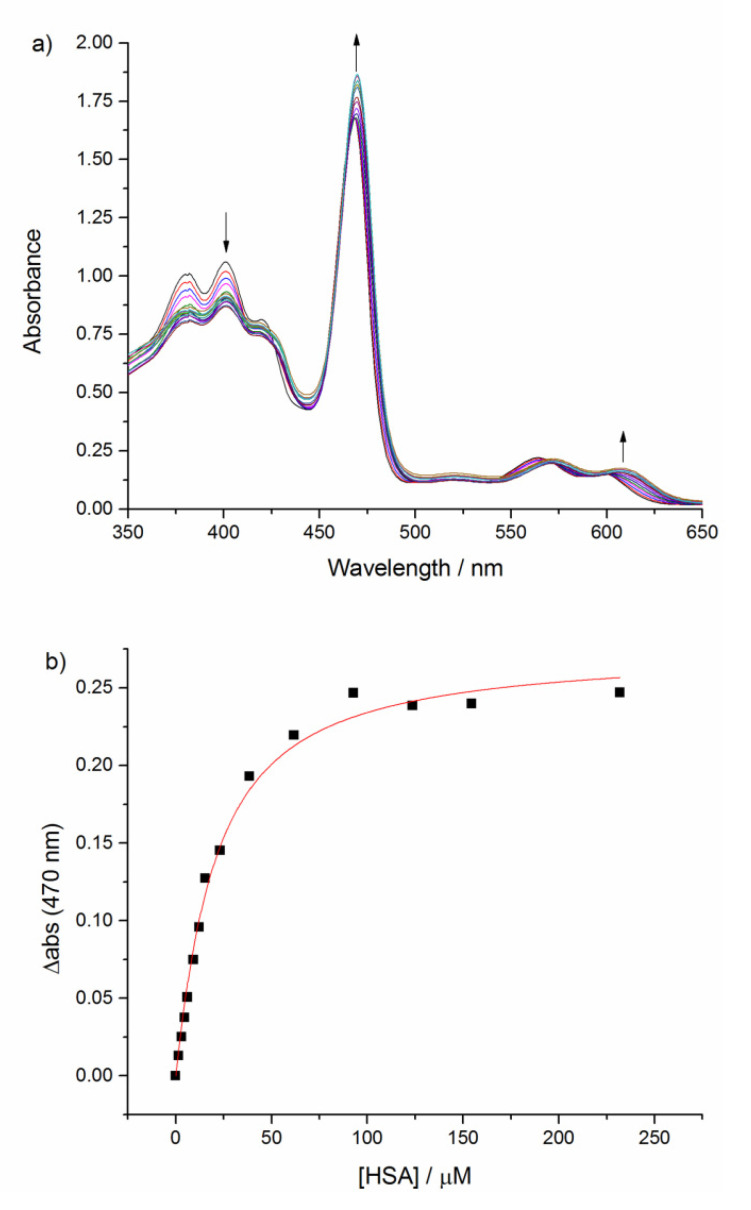
(**a**) UV absorbance spectra of 7.7 μM *m*-MnP2 titrated against 0–15 eq. of human serum albumin (has) in 25 mM pH 7.2 HEPES buffer at 25 °C. (**b**) The change in UV-Vis absorbance at 470 nm was plotted and fitted using OriginLab^®^ 9.0 to obtain a dissociation constant *K*_d_ of 16.5 ± 1.5 µM (see supporting information for details).

**Figure 4:**
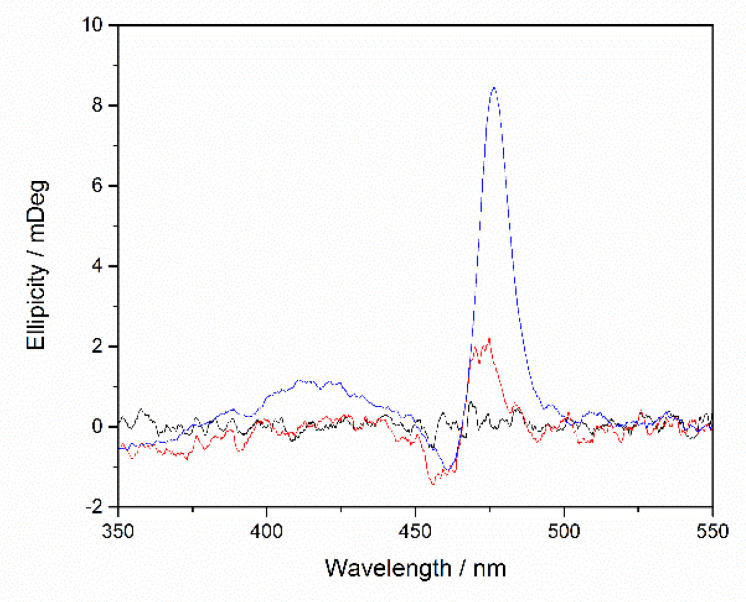
Circular dichroism (CD) spectra of 25 mM HEPES buffer (black), *m*-MnP2 with 1 eq. of HSA (red), and MnP2 with 1 eq. HSA (blue). Concentrations of MnPs are normalized to 3 μM.

The non-covalent binding of *m*-MnP2 with HSA is crucial to understanding its potential as a BPA. Thus, a UV-visible spectroscopy titration experiment was performed in order to monitor the protein binding and to quantify the dissociation constant *K*_d_. Upon titration of HSA into *m*-MnP2, a 2 nm redshift at the Soret band (468 nm) was observed (Figure 3a). Through fitting the absorbance change at 470 nm over the concentration of HSA, a relatively weak *K*_d_ of 16.5 ± 1.5 μM was obtained (Figure 3b). In comparison, MnP2 affinity to HSA has a *K*_d_ = 0.55 ± 0.26 μM. To further confirm the affinity of *m*-MnP2 towards HSA, a circular dichroism (CD) study was performed. If the *meta* analogue of MnP2 is able to bind specifically and tightly to the same binding pocket as MnP2, then its induced chirality would produce a strong ellipicity signal. Unlike the strong ellipticity induced by the MnP2 and HSA complex [14], the *m*-MnP2 and HSA complex yields a weak signal at 472 nm (Figure 4), preventing further quantitative study on protein affinity. The low CD signal of the *m*-MnP2–HSA complex further implied a less specific and weak affinity of *m*-MnP2 to HSA.

### 2.4. Relaxometry Studies with HSA

**Figure 5:**
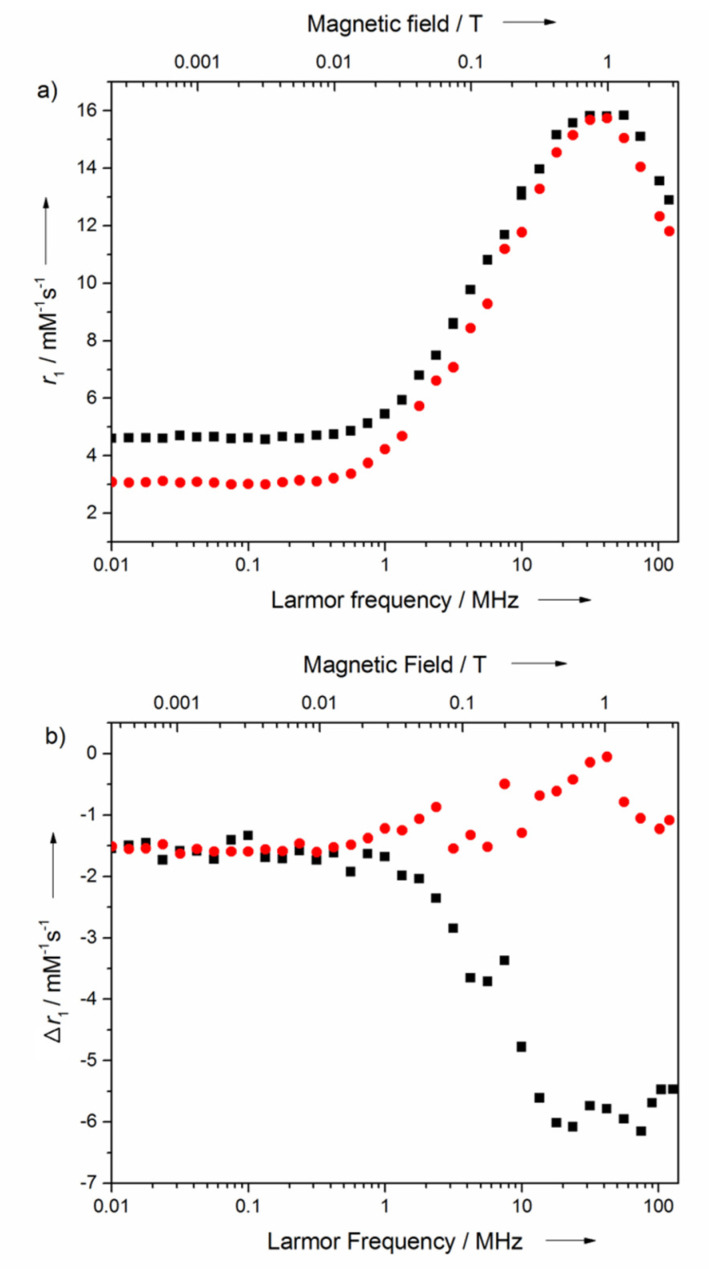
(**a**) NMRD profiles of free *m*-MnP2 (⬛) and *m*-MnP2 with HSA (🔴) in 24 mM pH 7.2 HEPES buffer measured at 25 °C from 0.01–128 MHz. (**b**) The change in *r*_1_ of *m*-MnP2 (🔴) and MnP2 (⬛) upon binding with HSA.

We next studied how binding with HSA influences the relaxivity of *m*-MnP2. The NMRD profile for *m*-MnP2 in the presence of 1 equivalence of HSA was obtained and compared with that of free *m*-MnP2. As shown in Figure 5a, the *r*_1_ values of *m*-MnP2 in general decreases upon HSA binding, but the magnitude of the change is field-dependent, mainly impacting the low fields. The *r*_1_ decrease (∆*r*_1_) caused by HSA binding is more significant between 0.01 to 0.3MHz, where the *r*_1_ is relatively constant (4.6 mM^−1^ s^−1^ for free *m*-MnP2, and 3.1 mM^−1^ s^−1^ for *m*-MnP2–HSA complex). As the field increases, the *r*_1_ difference becomes smaller. In contrast, at fields higher than 10 MHz, the *r*_1_ of the free and HSA bound *m*-MnP2 converge at 1 T (~15.8 mM^−1^ s^−1^). This change in *r*_1_ upon HSA binding is different from the NMRD profiles of MnP2, which shows a larger drop in relaxivity (∆*r*_1_) at both low and high magnetic fields upon HSA binding (Figure 5b).

**Figure 6:**
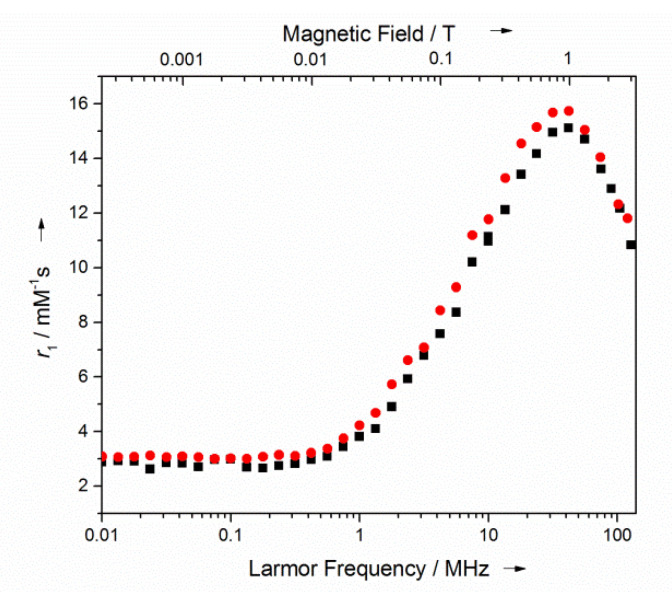
NMRD profiles of MnP2-HSA (🔴) and *m*-MnP2-HSA (⬛) in pH 7.2 25 mM HEPES at 25 °C, measured from 0.01–128 MHz.

Although MnP2 has higher *r*_1_ at high clinical fields compared to *m*-MnP2, the compromised *r*_1_ (∆*r*_1_) for MnP2 upon binding with HSA is much greater than that for *m*-MnP2 (Figure 5b). As a result, both agents are expected (and implied) to have very similar in vivo contrast enhancement efficacy, assuming similar pharmacokinetics. The similar NMRD profiles shown in Figure 6 suggest that, at low field, the relatively fast electron relaxation of MnPs makes electron relaxation time *τ*_e_ the dominant correlation time. At the high field, where electron relaxation slows down, the tumbling rate *τ*_R_ becomes the dominant factor. However, because the NMRD profile is governed by many interplayed factors, other possibilities that lead to this coincidence of similar NMRD cannot be excluded.

## 3. Materials and Methods

All reagents and solvents were of commercial reagent grade and were used without further purification except where noted. Pyrrole, benzaldehyde, and BF_3_·Et_2_O were obtained from Sigma-Aldrich. 3-boropinacolatobenzaldehyde was purchased from Boron Molecular. DDQ and Pd(PPh_3_)Cl_2_ were obtained from AK Scientific, Inc. (Union City, CA, USA) TBAF was purchased from Alfa Aesar. HEPES buffer (25 mM, pH 7.2) fixed at the ionic strength of 0.1 M using NaCl was used for all measurements. HSA was purchased from Sigma Chemical Co. (St. Louis, MO., USA) and its molecular weight was assumed to be 66,478 Da as specified. All other reagents and solvents were purchased from Caledon Laboratories, Ltd. (Georgetown, ON, Canada) TLC was performed using TLC silica gel 60 F_254_ plates from Merck KGaA. Column Chromatography was set up using Silica Gel 60, 250–400 microns from Desican Inc. (Scarborough, ON, Canada) Cation ion exchange was performed using an Amberlite^®^ IR120, H resin. Dialysis was performed with Sigma Aldrich Pur-A-Lyzer^TM^ Mega 3500 MWCO. All the spectroscopy data for structural characterizations were obtained using the research facilities at University of Toronto Scarborough campus (TRACES center) or at St. George campus (Chemistry Department). NMR spectra were recorded on Bruker-500 MHz or Varian Unity 500 MHz spectrometer. UV-Visible absorption spectra were recorded on an Agilent 8453 Spectroscopy System in a 1 cm path length cuvette at a scan rate of 4800 nm/min and data points were taken every 1 nm. Mass spectra were obtained from an ABI/Sciex Qstar mass spectrometer (ESI). CD spectra were obtained using Jasco J-815 Circular Dichroism Spectrophotometer. The CD spectrum was recorded in a 1 cm path length cuvette at a scan rate of 100 nm/min and data points were taken every 0.1 nm.

### Synthesis

5-(3-boropinaocolatophenyl)-10,15,20-triphenylporphyrin (**1**): The reaction was carried out in the dark under room temperature. 520 mL DCM, 1.3 g NaCl (22.2 mmol) and 2.68 g 3-boropinacolatobenzaldehyde (11.48 mmol) were stirred in a 1 L round bottom flask for 20 min under argon atmosphere. 3.2 mL pyrrole (45.98 mmol) and 3.52 mL benzaldehyde (34.83 mmol) were added and stirred for 10 more min. 0.52 mL BF_3_·Et_2_O (4.30 mmol) was then added dropwise. After 1 h of additional stirring, 7.84 g DDQ (36.12 mmol) was added to the mixture followed by 2h of stirring. The dark green crude solution was concentrated by rotary evaporation followed by filtration using a double-layer silica/alumina column, washed with DCM. The filtered solution was concentrated and purified by column chromatography on silica gel with elution solvent 7:3 DCM/hexanes. 380.0 mg (18%) of 1 was isolated as dark purple solid. ESI-MS (positive mode, DCM/Methanol, Figure S3) found *m*/*z* = 741.4 ([M + H]^+^), calculated for C_50_H_42_BN_4_O_2_^+^ (*m*/*z* = 741.34). ^1^H-NMR (500 MHz, CDCl_3_, Figure S1): δ (ppm) −2.79 (2H, s, NH), 1.39 (12H, s, methyl), 7.76 (10H, m, phenyl), 8.22 (7H, m, phenyl), 8.29 (1H, d, *J* = 8 Hz, sub Ph-p), 8.65 (1H, s, sub Ph-o’), 8.84 (8H, m, por-β).

3,3′-bis(5,10,15-triphenylporphyrin-20-yl)biphenyl (2): 22.2 mg of 1 (0.03 mmol) was added along with 2.2 mg Pd(PPh_3_)_2_Cl_2_ (0.003 mmol) and 10.9 mg TBAF (0.035 mmol) in a reaction flask. 2.68 mL of a 1:4 mixture of dH_2_O:THF was added as solvent. The reaction mixture was stirred in room temperature while opened to air. Reaction progression was monitored using TLC (1:1 DCM/hexanes). After 3 h of stirring, the reaction was stopped by extraction with DCM, in which the product was seen in the organic layer as a suspension instead of a solution. The organic layer was dried over Na_2_SO_4_ and concentrated via rotary evaporation. Repeated centrifugation (5000× *g*, 10 min at 25 °C) and resuspension of the pellet using 1:1 DCM/hexanes followed by the same procedure using diethyl ether was used to purify 2. The product was shown to be pure once the organic solvents were clear upon centrifugation. 2 was collected as a purple powder (10.3 mg, 56%). TLC was used to determine product purity (R_f_ = 0, 1:1 DCM/hexanes).

3,3′-bis(5,10,15-tris(4-sulfonatophenyl)porphin-20-yl)biphenyl (*m*-P2): 5 mg of 2 (0.0041 mmol) was dissolved in 0.7 mL of 18 M concentrated sulphuric acid. The solution was stirred at 55 °C for 5 h and quenched by neutralization with 0.1 M NaOH. The crude solution was dialyzed with a 3500 MWCO membrane to remove excess salt and purified by automated flash chromatography on a 15.5 g C18 Redisep^®^ R_f_ GOLD column using acetonitrile and 10 mM NH_4_OAc as the mobile phase, using a gradient sequence 0–3 CV: constant 25% acetonitrile; 3–12 CV: linear gradient from 25% to 50% acetonitrile; 12–16 CV: 50% to 100% acetonitrile at a flow rate of 35 mL/min. Purified product above 90% purity was collected and dried by lyophilisation. 2.6 mg of dark red solid (36%) of *m*-P2 was collected. Negative mode ESI-MS (H_2_O, Figure S4) found *m*/*z* = 283.6 ([M]^6−^), 340.4 ([MH]^5−^), 425.8 ([MH_2_]^4−^), calculated for C_88_H_48_N_8_O_18_S_6_^6−^ (*m*/*z* = 283.4), C_88_H_49_N_8_O_18_S_6_^5−^ (*m*/*z* = 340.3), C_88_H_50_N_8_O_18_S_6_^4−^ (*m*/*z* = 425.6). ^1^H-NMR (500 MHz, DMSO-*d*_6_, Figure S2): δ (ppm) −2.90 (4H, s, NH), 7.94 (2H, t, *J* = 8 Hz, bridge Ph-*m*), 8.05 (12 H, m, Ph-*m*), 8.18 (12H, m, Ph-*o*), 8.26 (2H, d, *J* = 7 Hz, bridge Ph-*p*), 8.43 (2H, d, *J* = 8 Hz, bridge Ph-*o*), 8.78 (2H, s, bridge Ph-*o*’), 8.85 (m, 12H, por-β), 8.99 (4H, d, *J* = 8 Hz, por-β). The extinction coefficient of *m*-P2 is 480 000 M^−1^ cm^−1^ at 422 nm, obtained from UV-Visible measurements in 25 mM pH 7.2 HEPES buffer based on dry mass.

3,3′-bis-Mn(III)5,10,15-tris(4-sulfonatophenyl)porphin-20-yl)biphenyl (*m*-MnP2): 5.0 mg *m*-P2 (0.0041 mmol), 11.3 mg Mn(OAc)_2_ (0.065 mmol) and 6 μL DIPEA (0.034 mmol) were stirred in 0.8 mL DMF for 4 h under reflux and monitored via UV-Visible spectroscopy. The crude was filtered to remove excess Mn(II) salts by a silica column using pure DMF as the eluent followed by dialysis using a 3500 MWCO membrane. To remove the remaining trace Mn(II) ions, an Amberlite IR120 cation exchange column (sodium form) was used to yield 7.12 mg (91%) dark green powder. ESI-MS of *m*-MnP2 (negative mode, H_2_O, Figure S5) found *m*/*z* = 451.80 ([M]^4−^), calculated for C_88_H_48_N_8_O_18_S_6_Mn_2_^4−^ (*m*/*z* = 451.5). The extinction coefficient of *m*-MnP2 is 212 000 M^−1^ cm^−1^ at 468 nm, obtained from FAAS and UV-Visible measurements in 25 mM pH 7.2 HEPES buffer.

## 4. Conclusions

An intermediate size MnP dimer, *m*-MnP2, was designed with a twisted biphenyl linker for the investigation of its field dependent relaxivities and interaction with HSA. The molar *r*_1_ of *m*-MnP2 peaked at 1 T (31.6 mM^−1^ s^−1^) and extended to higher fields up to ~3 T (120 MHz) with moderate decrease (25.8 mM^−1^ s^−1^), favouring high *r*_1_ at high magnetic fields. Relative to MnTPPS and MnP2, the intermediate size of *m*-MnP2, determined by geometric flexibility, is well correlated to their respective relaxivities at the 1 T region. HSA titration studies monitored by UV-visible and CD spectra indicated *m*-MnP2 formed a non-covalent HSA complex, with the interaction tuned down to be less strong in contrast to MnP2, suggesting the modification on biphenyl bridge indeed impacts the interaction with HSA. The *m*-MnP2–HSA complex exhibited weaker binding in comparison to MnP2 but the *r*_1_ of both HSA complexes are comparable, especially at magnetic fields above 1 T, thus exhibiting higher sensitivities than the regular Gd agents for high field applications. Therefore, the *m*-MnP2–HSA complex is expected to serve as a high relaxivity BPA with potential shorter vascular retention time and possible partial renal clearance, which will be verified by in vivo studies in the near future. The molecular principle demonstrated in this work will guide the design of future modulations of next generation BPA based on porphyrins.

## Figures and Tables

**Table 1:** *r*_1_ of free MnP2 and *m*-MnP2 at clinical magnetic fields normalized per Mn, measured in 25 mM pH 7.2 HEPES at 25 °C.

	*r*_1_ at 1.6 T (mM^−1^ s^−1^)	*r*_1_ at 3 T (mM^−1^ s^−1^)
MnP2	20.2	14.7
*m*-MnP2	15.4	12.9

## Supplementary Materials

Supplementary information is available online at https://www.mdpi.com/1424-8247/13/10/282/s1. Figure S1. ^1^H-NMR of 1; Figure S2. ^1^H-NMR of *m*-P2; Figure S3. Mass spectrum of 1; Figure S4. Mass spectrum of *m*-P2; Figure S5. Mass spectrum of *m*-MnP2; Figure S6. UV-Visible spectra of *m*-P2 and *m*-MnP2; Figure S7 Molecular dynamics calculations for both MnP2 dimers; Figure S8 Mass spectrum of oversulfonated *m*-P2;
