# Supplementary material for: A Second Generation Mn-Porphyrin Dimer with a Twisted Linker as a Potential Blood Pool Agent for MRI: Tuning the Geometry and Binding with HSA
Source: Pharmaceuticals (Basel). 2020 Sep 29;13(10):282. doi: 10.3390/ph13100282 (PMC7599881; doi:10.3390/ph13100282)
Supplement: Supplementary file 1 [file pharmaceuticals-13-00282-s001.pdf]

A Second Generation Mn-Porphyrin Dimer with a Twisted Linker as a Potential Blood Pool Agent for MRI: Tuning the Geometry and Binding with Serum Albumin

Supporting Information

Table of Contents

Fig. S.1. ^{1}H-NMR of **1**	2
Fig. S.2. ^{1}H-NMR of *m*-P2	3
Fig. S.3. Mass spectrum of **1**	4
Fig. S.4. Mass spectrum of *m*-P2	5
Fig. S.5. Mass spectrum of *m*-MnP2.	6
Fig. S.6. UV-Visible spectra of *m*-P2 and *m*-MnP2.	7
Fig. S.7. Molecular dynamics calculations for both MnP2 dimers	8
Fig. S.8. Mass spectrum of oversulfonated *m*-P2	9
Dissociation constant determination	10

Characterization

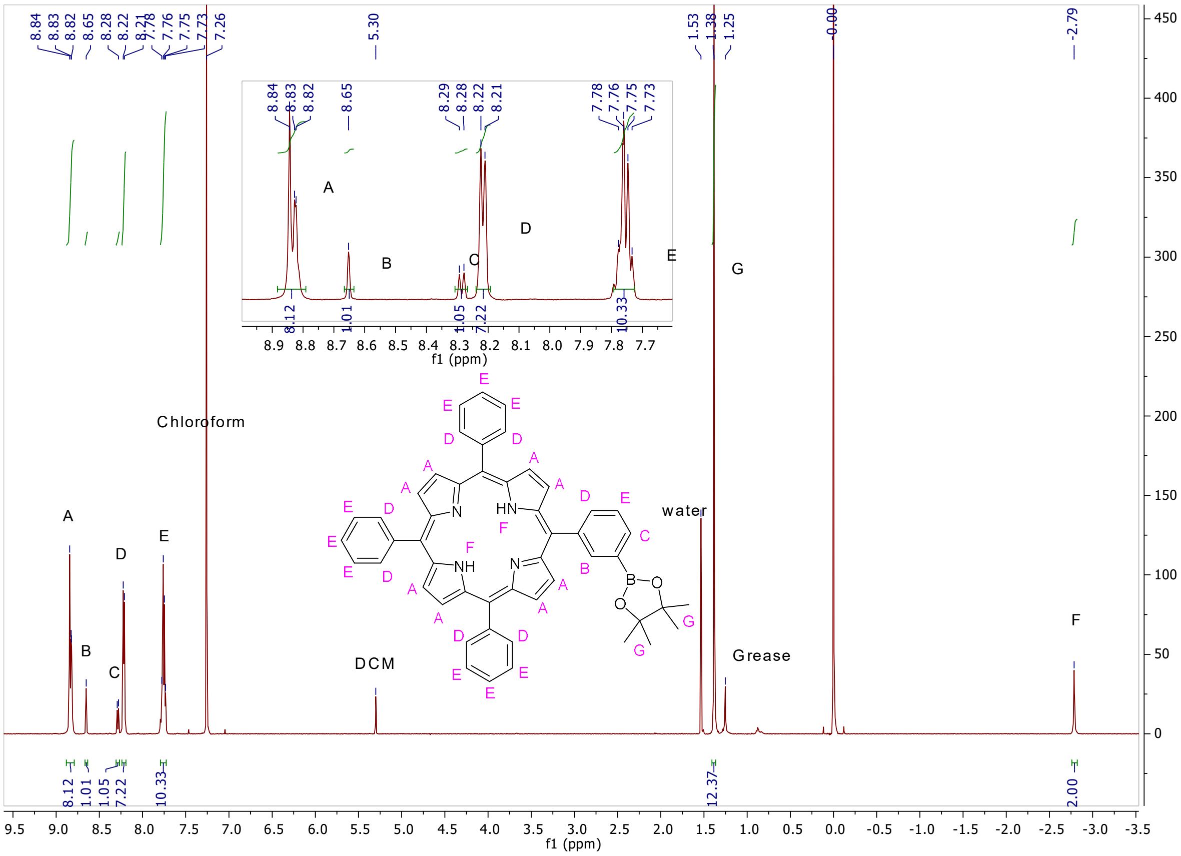

Figure S1. ^{1}H-NMR of **1** acquired in $CDCl_3$ with 0.1% TMS. Residual solvent peaks are $CHCl_3$, CH_2Cl_2, grease, and water.

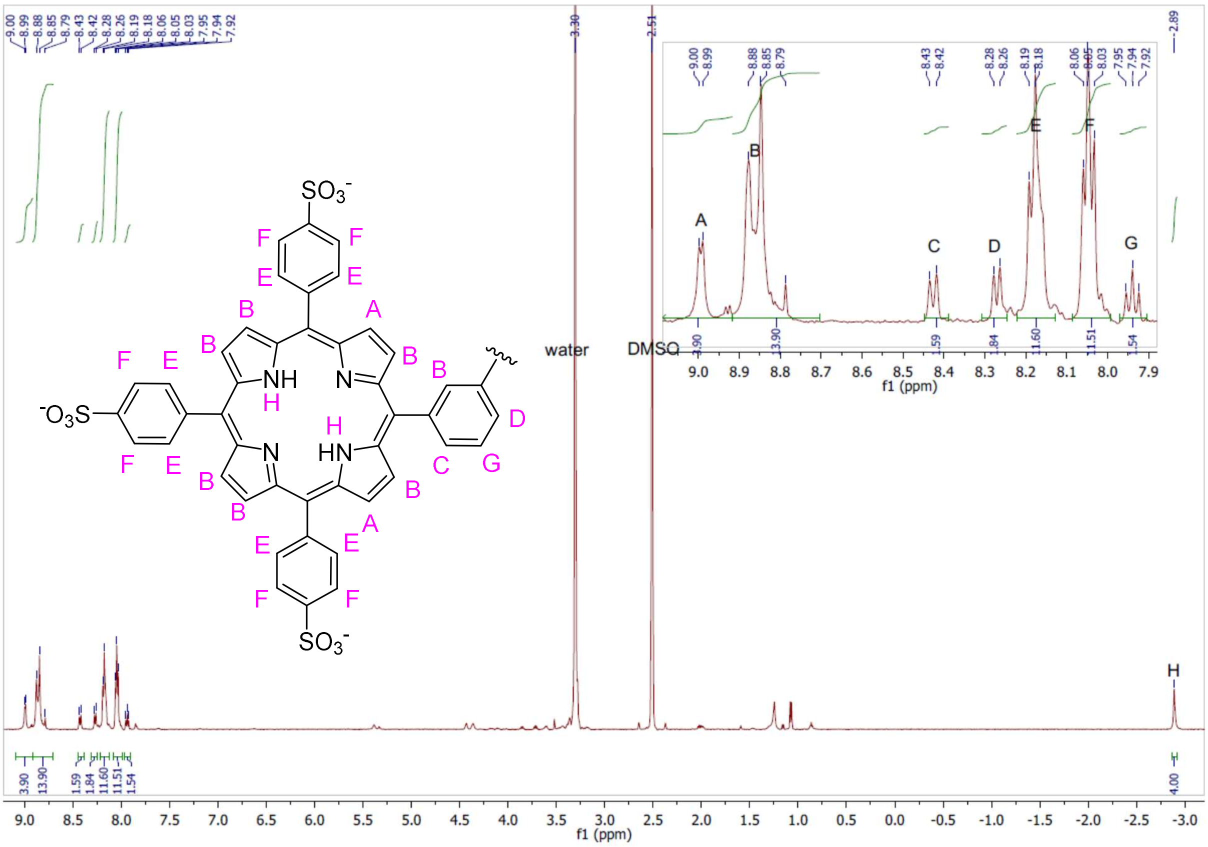

Figure S2. ^{1}H-NMR of *m*-P2 acquired in DMSO-d_6. Residual solvent peaks are DMSO and water.

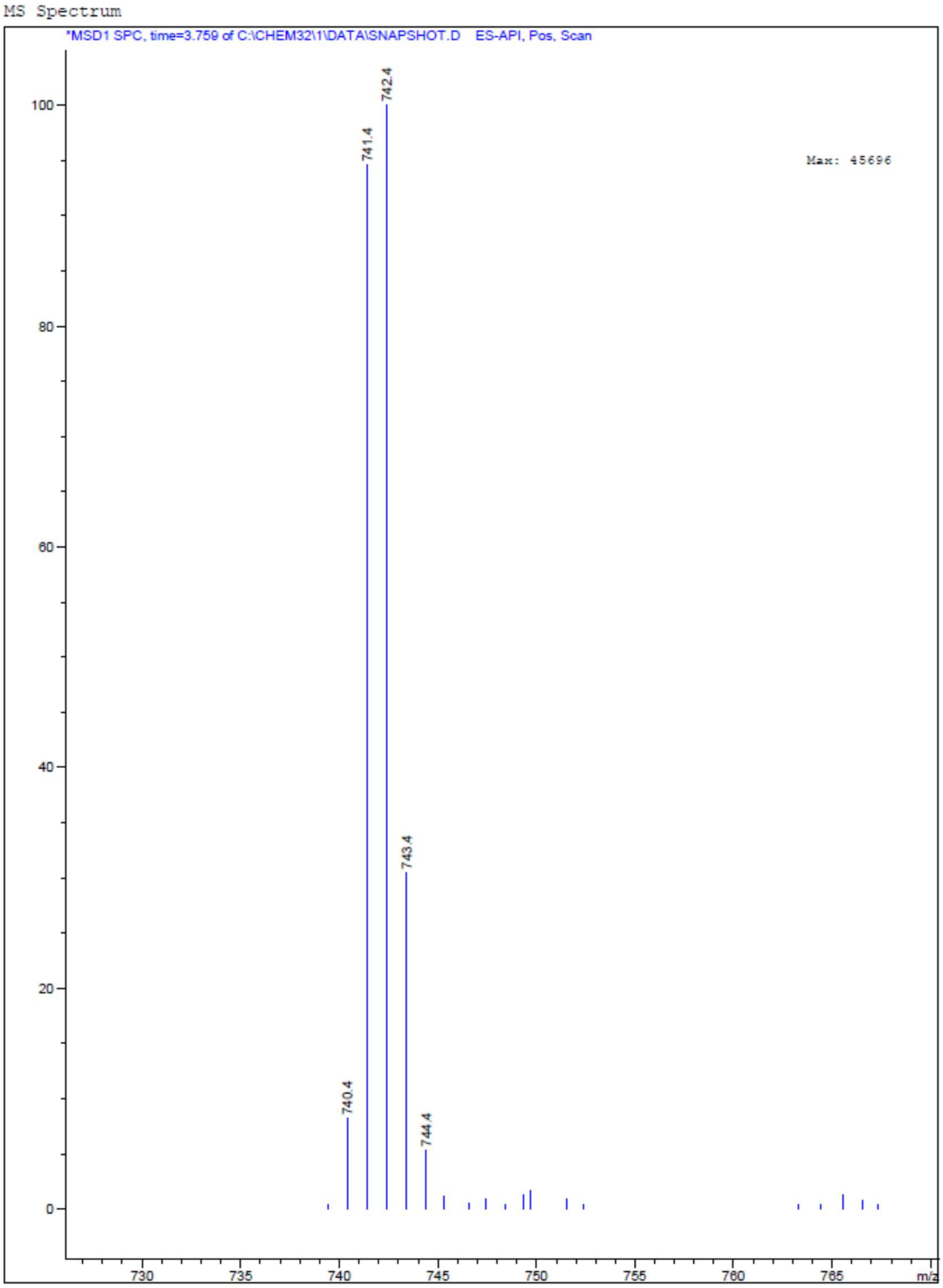

Figure S3. Positive mode ESI-MS of **1**.

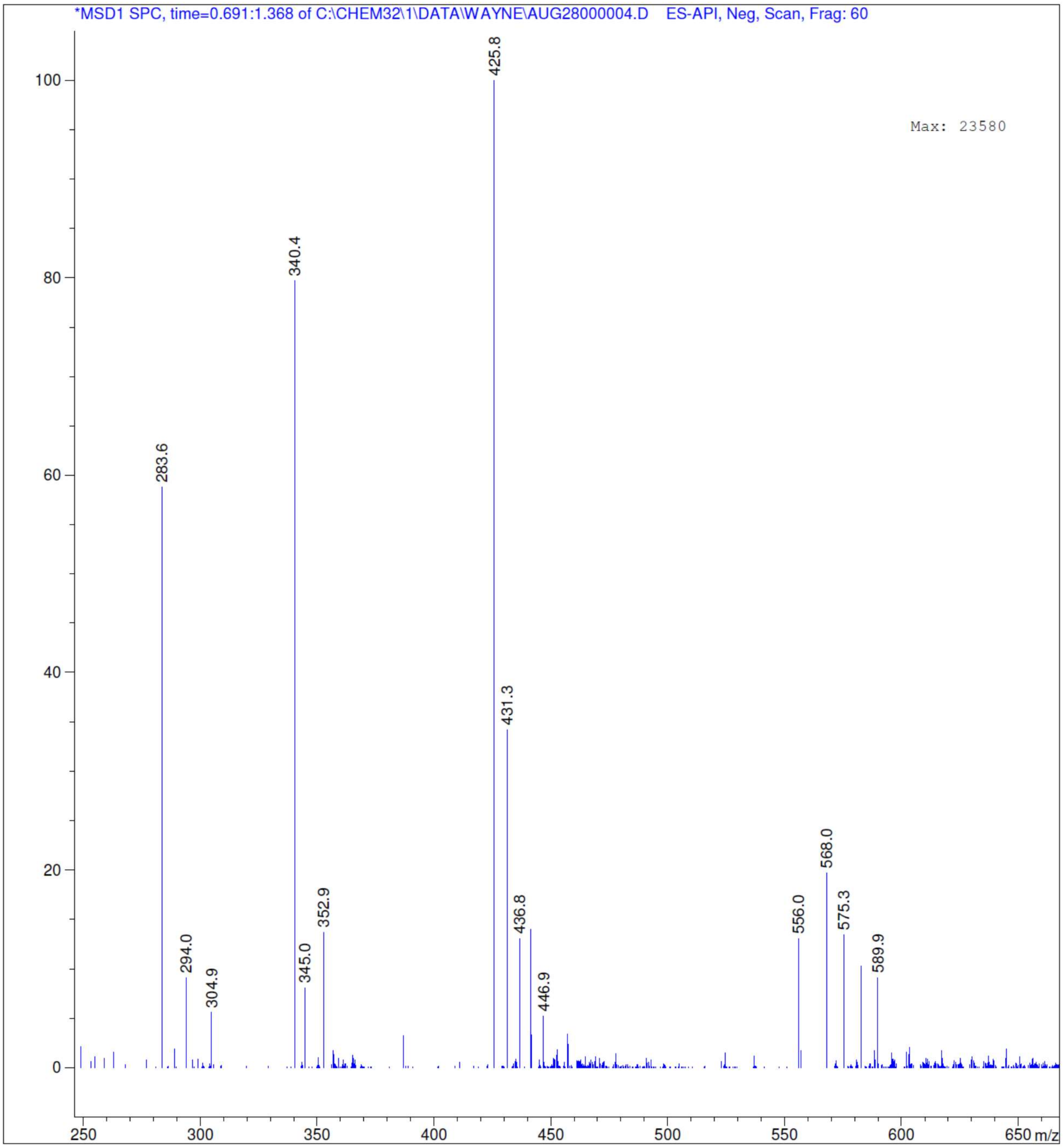

Figure S4. Negative mode ESI-MS of *m*-P2.

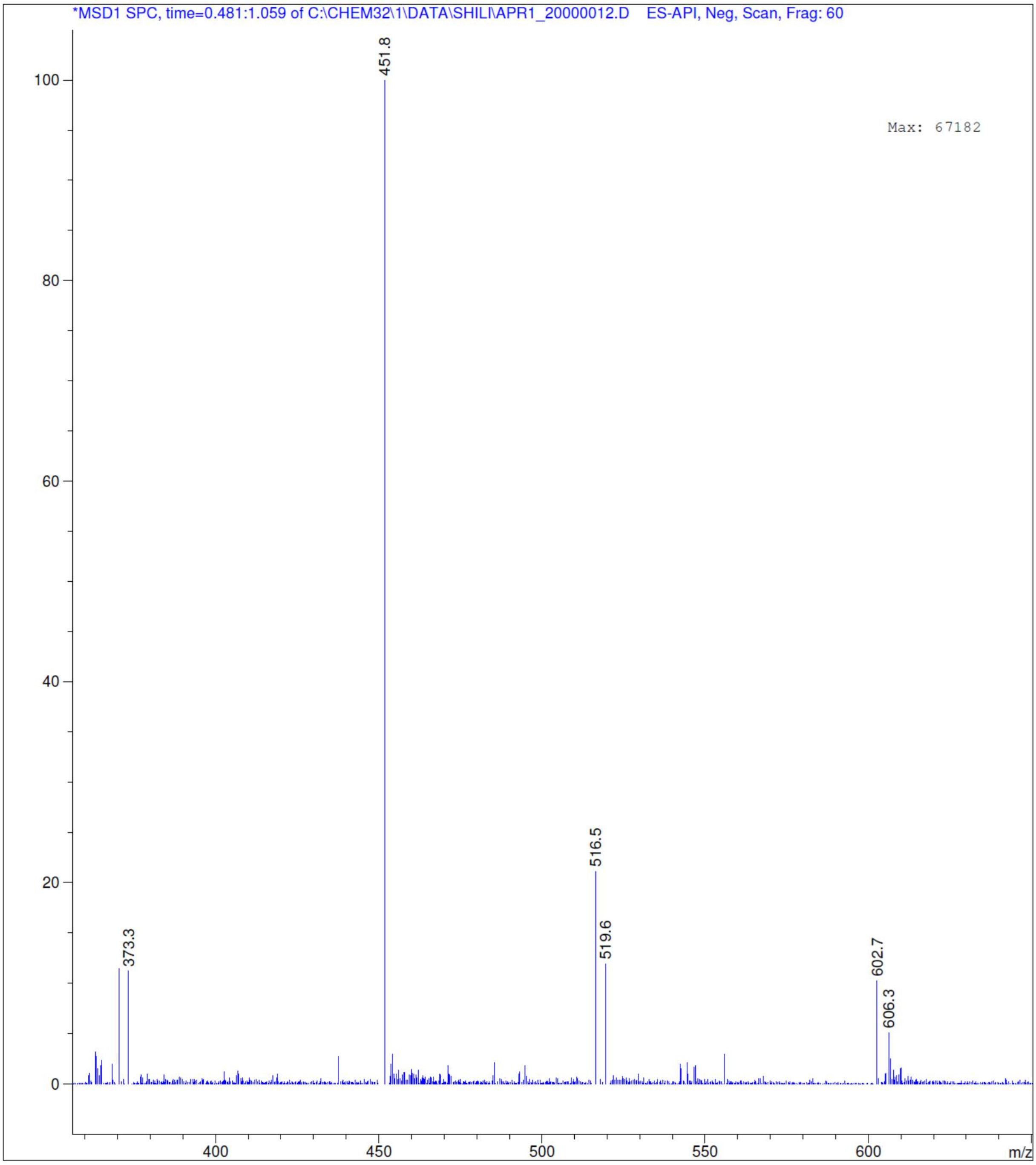

Figure S5. Negative mode ESI-MS of *m*-MnP2.

-

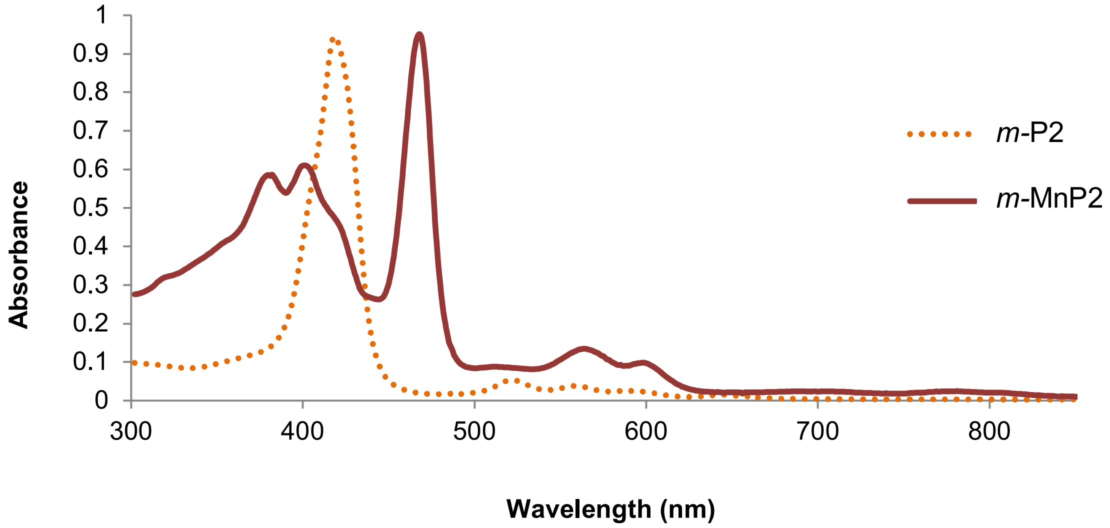

Figure S6. UV-Visible spectra of *m*-P2 and *m*-MnP2 measured in 25 mM pH 7 HEPES buffer. λ_{max} of *m*-P2 = 422 nm, λ_{max} of *m*-MnP2 = 468 nm.

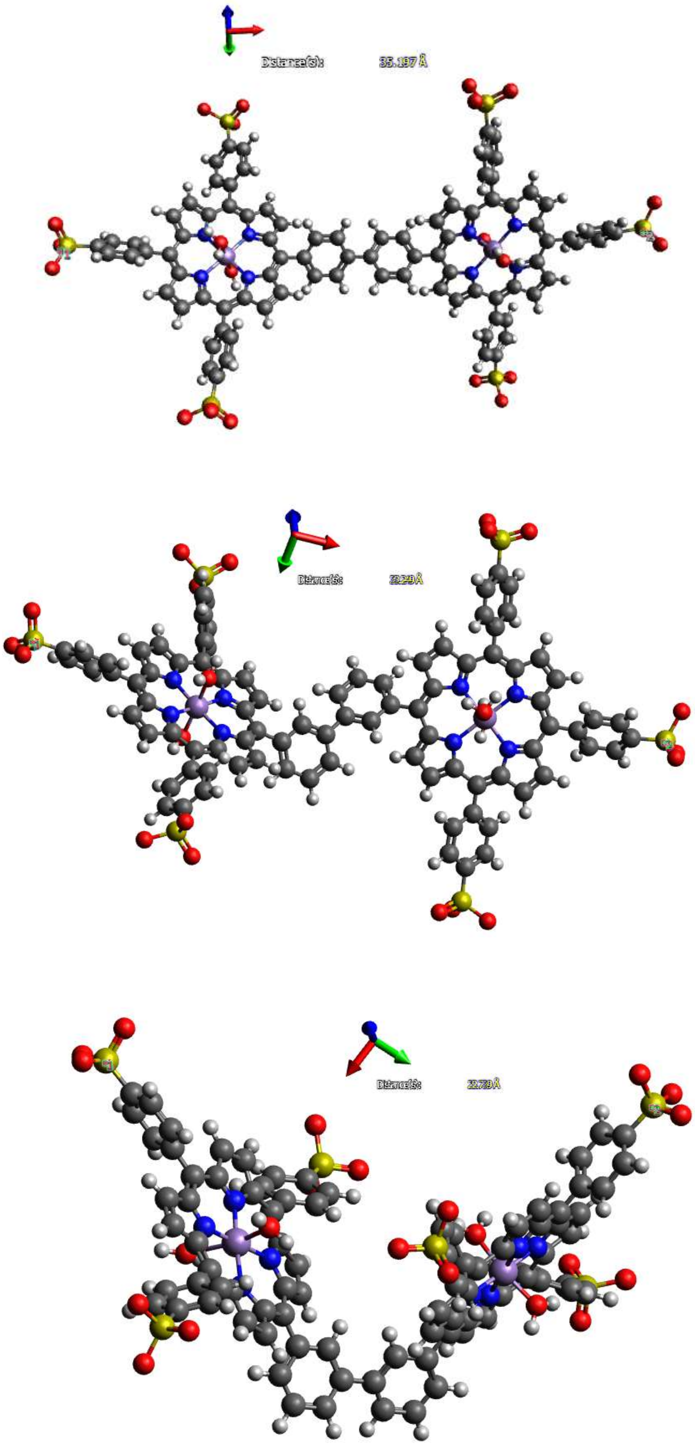

Figure S7. Molecular modeling of MnP2 (top) and two conformers of *m*-MnP2 (middle and bottom). The distance between two distal S-atoms are labeled. (35.197, 33.249 and 22.739 Å, respectively).

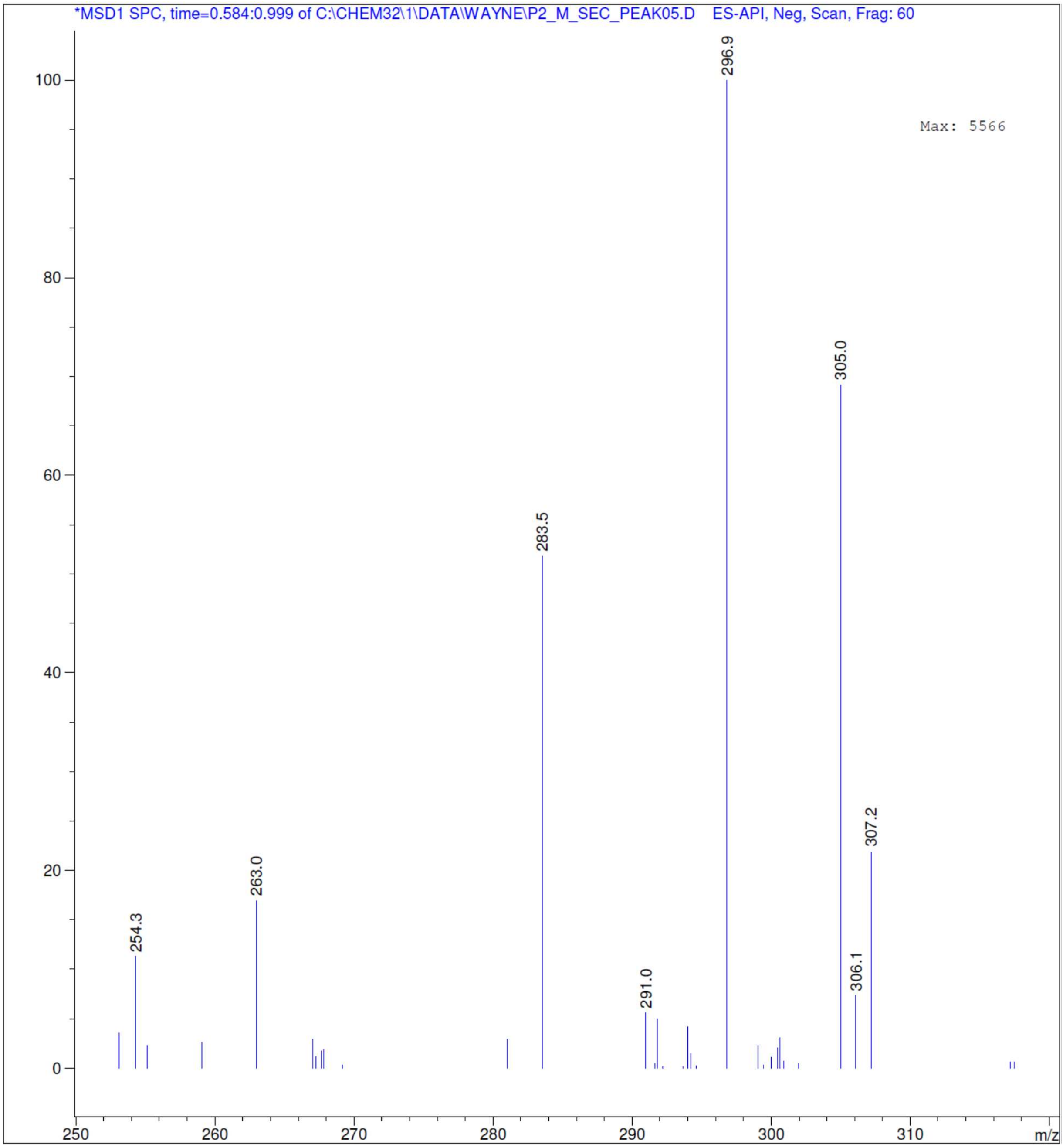

Fig. S8. Negative mode ESI-MS of oversulfonated *m*-P2 found *m/z* = 296.80 ($[M]^{6-}$), calculated for $C_{88}H_{49}N_8O_{21}S_7^{6-}$ (*m/z* = 296.69).

Dissociation Constant Determination

The dissociation constant, K_d, was obtained using the GraphPad Prism/OriginLab Pro 9.0 software by fitting the experimental data to the following equations:

$$\mathrm{LR} \overset{K_d}{\leftrightarrow} \mathrm{L} + \mathrm{R} \quad [1]$$

$$\mathrm{LR} = \frac{(x+L_0+K_d)-\sqrt{(x+L_0+K_d)^2-4x\times L_0}}{2} \quad [2]$$

$$\mathrm{L} = \mathrm{L}_0 - \mathrm{LR} \quad [3]$$

$$Y\text{-}Y_0 = M\mathrm{LR} \times \mathrm{LR} \quad [4]$$

These equations are based on the assumption that the porphyrin, L, and the HSA, R, are bound to form a 1:1 LR complex. L_0 is the total concentration of porphyrin and $\boldsymbol{x}$ denotes the total concentration of HSA in the solution. The *M*LR is the molar absorbance of the LR complex. *Y* and Y_0 are the observed absorbance and the initial absorbance respectively.
